# An SDR-Based Real-Time Testbed for GNSS Adaptive Array Anti-Jamming Algorithms Accelerated by GPU

**DOI:** 10.3390/s16030356

**Published:** 2016-03-11

**Authors:** Hailong Xu, Xiaowei Cui, Mingquan Lu

**Affiliations:** Department of Electronic Engineering, Tsinghua University, Weiqing Building, Tsinghua, Beijing 100084, China; xuhl07@163.com (H.X.); lumq@mail.tsinghua.edu.cn (M.L.)

**Keywords:** Global Navigation Satellite System (GNSS) anti-jamming, adaptive array, software-define radio (SDR), Space-Time Adaptive Processing (STAP), Space-Frequency Adaptive Processing (SFAP), nulling, beamforming

## Abstract

Nowadays, software-defined radio (SDR) has become a common approach to evaluate new algorithms. However, in the field of Global Navigation Satellite System (GNSS) adaptive array anti-jamming, previous work has been limited due to the high computational power demanded by adaptive algorithms, and often lack flexibility and configurability. In this paper, the design and implementation of an SDR-based real-time testbed for GNSS adaptive array anti-jamming accelerated by a Graphics Processing Unit (GPU) are documented. This testbed highlights itself as a feature-rich and extendible platform with great flexibility and configurability, as well as high computational performance. Both Space-Time Adaptive Processing (STAP) and Space-Frequency Adaptive Processing (SFAP) are implemented with a wide range of parameters. Raw data from as many as eight antenna elements can be processed in real-time in either an adaptive nulling or beamforming mode. To fully take advantage of the parallelism resource provided by the GPU, a batched method in programming is proposed. Tests and experiments are conducted to evaluate both the computational and anti-jamming performance. This platform can be used for research and prototyping, as well as a real product in certain applications.

## 1. Introduction

Due to the vulnerability of Global Navigation Satellite System (GNSS) signals to radio frequency (RF) interferences, anti-jamming design has always been an important issue in both military and civilian applications. In order to increase the resistance of GNSS receivers to intentional and unintentional interferences, adaptive array processing techniques such as space-time adaptive processing (STAP) and space-frequency adaptive processing (SFAP) are widely used. Compared to space adaptive processing (SAP), they exploit both temporal (frequency) and spatial domain, providing more degrees of freedom (DOF) [1]. Previous literature shows that wide-band and narrow-band interference can be mitigated effectively through these algorithms.

Jamming and anti-jamming are best considered together. An anti-jamming algorithm with certain parameters may perform quite differently while the jamming pattern varies. It is necessary to study the relation, so that we can choose the most appropriate anti-jamming algorithm with the most appropriate parameters against specific interference scenarios. Although software tools such as MATLAB can be used to simulate on the principle level, it is quite far from the actual physical environment, reducing the validity of the results. It is hoped that different adaptive array algorithms can be tested in real-time in real interference scenarios, while the parameters can be flexibly configured in the field. In order to meet this demand, a GNSS adaptive array anti-jamming testbed with a high level of flexibility and configurability is designed and implemented in this paper.

Adaptive array algorithms are very computational costly, so traditionally they are implemented on hardware such as Field Programmable Gate Arrays (FPGAs) or Application Specific Integrated Circuits (ASICs) to meet the real-time requirement. However, this hardware implementation approach has some drawbacks and is not suitable for our testbed. Firstly, implementing on hardware requires a long development cycle, and is difficult to update and customize. Secondly, once the program is compiled and burned into the hardware, algorithms and parameters are difficult to be configured. Thirdly, it is hard to observe any intermediate states while the algorithm is running. On the contrary, a software-defined approach makes up all the short-comings above. It has greater flexibility, and can provide rich functionality. Using high-level programming languages such as C++ and C#, many libraries can be used, making the development process much faster. Different algorithms and parameters can be integrated into one implementation, making it a fertile ground for researchers to explore more possibilities. The real-time display of intermediate states by the graphic user interface (GUI) also makes assessing and verifying the algorithms more direct and timely. In summary, a software defined approach is preferred in the design and implementation of our testbed.

While a software implementation exceeds a hardware implementation in many ways such as flexibility, configurability and cost, special attention should be paid to satisfy the real-time requirement, especially in applications with a high data throughput. Adaptive array anti-jamming algorithms such as STAP and SFAP include a large number of matrix multiplication, linear equations solving and other complex operations. The amount of calculation is so high that even a modern Central Processing Unit (CPU) with many cores has difficulties to run them in real time. However, Graphics Processing Units (GPUs) have achieved great success in general-purpose computing in the last ten years. Unlike a CPU which has 4 cores or 8 cores with most transistors devoted to data caching and flow controlling, a GPU generally has hundreds of cores with most transistors devoted to data processing. Tens of thousands of threads can run concurrently on a single GPU, providing great speed-up against CPU in computational intensive applications. So in this project a GPU is selected as an accelerator.

Using a GPU as an accelerator for GNSS software-defined receivers has been studied intensively in the past ten years [2,3,4,5]. One of the most representatives is the STARx developed by a group of researchers in Tsinghua University, China [6]. By putting the acquisition search and correlation operations on the GPU, the receiver is able to process all civil signals from all GNSS systems in real time. Despite that software receivers have become a common way to evaluate signals and test new algorithms nowadays, work in GNSS adaptive array anti-jamming field to employ a software-defined approach is still limited, due to the high data throughput and computational complexity of adaptive array algorithms. As far as the authors know, the two most updated works in open literature are contributed by a group of researchers in Stanford University [7,8]. In [7] a real-time GPS receiver with adaptive beam-steering capability using a software-defined approach based on a quad-core CPU coupled with a GPU is developed. This receiver offers sufficient computational capability to support a four-element antenna array to form 12 beam-steering channels for all tracking satellites in view using SAP, and processes digital samples of GPS L1 Coarse/Acquisition (C/A) and L5 signals at a 40 MSPS rate with 14-bit resolution. After that, a CPU-based real-time beamforming receiver was implemented by the same group of researchers [8]. This receiver extends the function of the previous one by implementing the STAP algorithm. Single Instruction Multiple Data (SIMD) instructions assembly coding and multithreaded programming are used to reduce computational complexity. However, both of the two receivers are especially developed for beamforming and are limited in flexibility and configurability. Furthermore, they both exploit the iterative Least Mean Square (LMS) method to reduce computational complexity by avoiding the matrix inversion operation. Distinguished from the above two works, our testbed in this paper is for anti-jamming algorithms including not only SAP and STAP, but also SFAP, towards both adaptive nulling and beamforming. The Sample Matrix Inversion (SMI) method which requires matrix inversion operations is implemented. Special efforts are made to provide a high configurability of algorithms and parameter, as well as a high computational performance, which makes our testbed to exceed work in previous literature. A comparison between the previous works and our testbed is summarized in Table 1.

The above capabilities of our testbed are attributed to the GPU programs to a great extent. Although massive parallelism can be provided by GPU, it is the programmers’ duty to map algorithms properly to the hardware architecture in order to fully take advantage of the parallel computational resources. This is often a tricky task, which contributes to the fact that until now, GPU programming is still highly application-relevant. In consideration of this, the GPU programming details are fully documented for reference. In particular, a batched method which allocates the computing tasks more efficiently on GPU is highlighted. It aims to calculate many small problems concurrently rather than serially. The batched method has been proposed in previous work [9,10], but has not been used in GNSS anti-jamming applications. In addition, Compute Unified Device Architecture (CUDA) codes are carefully tuned to achieve the largest possible speedup. Programming techniques are introduced with great detail.

The rest of this paper is organized as follows: first, the system design is outlined, with emphasis on the anti-jamming processing unit on which the software runs. Then, Section 3 gives a brief review of the STAP and SFAP algorithms, which is a requisite for the following contents. After that, the software architecture and GPU programming techniques are introduced in detail in Section 4. To show that the software implementation meets the real-time requirement very well, test results of computational performance are given in Section 5. In Section 6, experiments are conducted in real environment with interferences to validate the algorithms, while Section 7 concludes this paper.

## 2. System Design

In this section, the overall architecture and data flow path of the platform are outlined, including the hardware modules as well as the general purpose processors in which the software are embedded. In the system design, special attention is paid to adapt the high data throughput and computational complexity.

### 2.1. Platform Architecture and Data Flow

**Figure 1:**
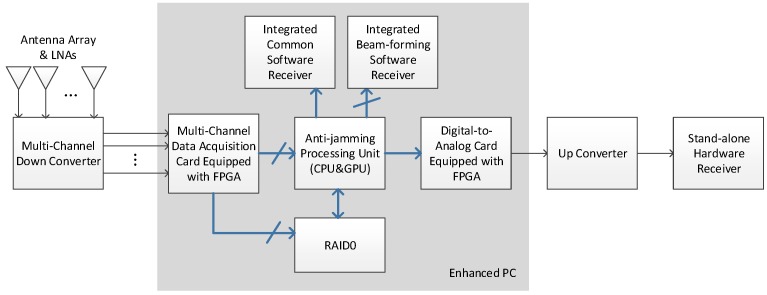
System architecture and data flow path of the adaptive array anti-jamming testbed. The thin black lines and arrows indicate the analog signal stream, while the bold blue lines and arrows indicate the digital data stream. The short slashes upon the lines indicate that the stream contains multiple channels.

**Figure 2:**
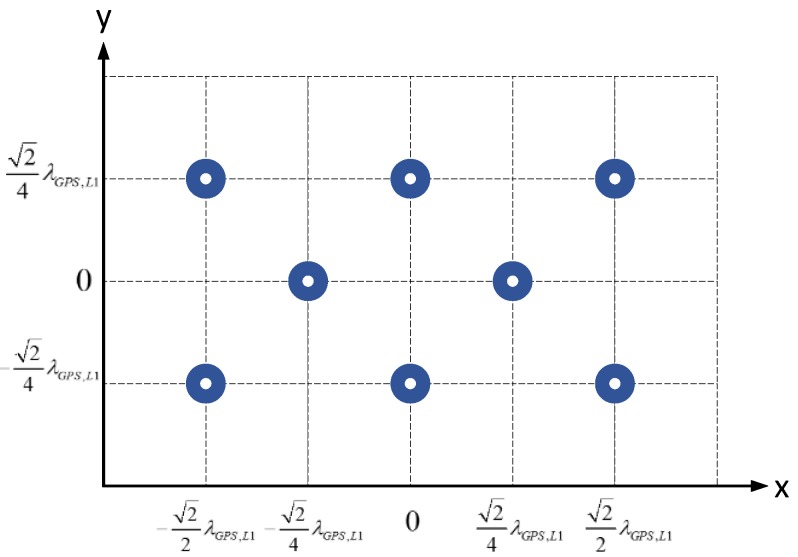
Geometry of the dual-frequency antenna array for GPS L1 and BDS B3 signals. The distance between the two nearest antenna elements is one half of the GPS L1 carrier wavelength.

As Figure 1 shows, the anti-jamming testbed performs the signal transforming and processing from the antenna array all the way to the GNSS receiver. In this paper we mainly focus on signals of GPS L1 C/A and China’s Beidou System (BDS) B3, which are two representatives of a low-chip-rate signal with 2 MHz bandwidth and a high-chip-signal with 20 MHz bandwidth respectively. At the very beginning, the antenna elements transform the radio signals in L-band to voltage signals, after which the low noise amplifiers (LNAs) amplify the signals with a gain of about 30 dB. Figure 2 shows the geometry of the specially designed planar antenna array with 8 elements. The objective of the design is that we can select different elements to form multiple types of array geometry with the number of antenna elements varying from 4 to 8. The square patch antennas of the array are dual-band, with the frequency centered at GPS L1 (1575.42 MHz) and BDS B3 (1268.52 MHz), respectively. Alternatively, a third-party antenna array and LNAs can also be used, as long as the element number is 8 or less.

Afterwards, the multi-channel down converter transforms the L-band signal to intermediate-frequency (IF) of about 45 MHz. The bandwidth of the RF front-ends is 2 MHz for GPS L1 C/A signal and 20 MHz for BDS B3 signal, respectively. The multi-antenna signals are then digitalized by a data acquisition card, which consists of 8 analog-to-digital converters (ADCs) and an FPGA whose framework logic can be modified. The card operates at a sampling rate of 60 MSPS to avoid spectrum aliases. The quantization resolution is 14 bits, thus enough ADC dynamic range is provided to support intensive jamming scenarios, although 2 bits are enough for a common receiver [11]. For the conversion to in-phase and quadrature (I/Q) signals as well as further reducing the data throughput, the 60 MSPS real data are then processed by the FPGA logic and transformed to 20 MSPS complex data. Performing the conversion to I/Q digitally also avoids the imbalance of analog I/Q circuits [12]. Then the output 20 MSPS multi-antenna complex data are transferred to the CPU and GPU for anti-jamming processing.

The anti-jamming processing unit is the computing core of the whole system and can work in two modes, namely the nulling mode and the beamforming mode. In the nulling mode, the multi-antenna digital complex data from the ADC card are transferred to the GPU for adaptive nulling processing. After the interferences are mitigated, the output single-channel complex data are transferred to the digital-to-analog converting (DAC) card, in which they are converted to IF analog signal. The signal is then up-converted to L-band before transferred to a stand-alone hardware receiver. As an alternative, the output data of the anti-jamming processing unit can also be directly transferred through the GPU memory to an integrated common software receiver, which is a modified version of STARx [6]. The word “common” is used to distinguish the single-input-channel receiver from a beamforming receiver which has multiple input channels.

In the beamforming mode, the anti-jamming processing unit uses the elevation and azimuth information of each visible satellites from the software receiver to form a dedicated beam in the antenna radio pattern towards each satellite. The multi-beam complex data are then transferred to the integrated beamforming software receiver. The feedback path of the elevation and azimuth information is not depicted in the figure, for its data bandwidth is small compared with the data stream.

Besides working in real-time mode, the testbed can also run in a post-processing mode, in which the input multi-antenna data of the anti-jamming processing unit are read from a file. These data are previously saved to the hard disk from the data acquisition card in a data fetching and logging process. If an eight-element antenna array is used, the data throughput to the hard disk can be as high as 80 × 8 = 640 MB/s, making data storage bandwidth the bottleneck of the whole procedure. Thus, a RAID hard disk array is used, as Figure 1 shows [13].

### 2.2. Anti-Jamming Processing Unit

A hybrid computing architecture is used in the anti-jamming processing unit, which comprises an Intel i7 CPU with four 2.5 GHz cores, and an NVidia GeForce GTX 770 GPU. The CPU is responsible for light sequence tasks, while the GPU is responsible for heavy parallel tasks.

GeForce 770 is NVidia’s consumer level product designed for gamers, providing a high computing capacity at a reasonably low cost. It has 1536 CUDA cores with a base clock of 1046 MHz. The memory is as much as 2 GB memory with a speed of 7 Gbps. This GPU is not so advanced compared to the following products such as GTX 980 and TITAN X, but it will be seen that with the programming optimization techniques in this paper, the capability of this GPU is enough to satisfy the real-time requirement.

In practice, the data acquisition card, the DAC card, the disk array, and the GPU are all integrated into an enhanced PC and transfers data through the PCI-E bus. The common software receiver and the beamforming software receiver runs on the anti-jamming processing unit.

## 3. Adaptive Array Anti-Jamming Algorithms

Both STAP and SFAP have been well studied and documented in [14,15,16], so we just give a brief review of them for reference. Meanwhile, the parameters and their range of values which we implemented in this project are emphasized. Implementations of the algorithms will be introduced in detail in the next section.

Throughout this paper, the following notation is adopted: small bold letters stand for vectors and capital bold letters stand for matrices, while superscripts H and T denote conjugate transpose and transpose respectively.

### 3.1. Spatial-Time Adaptive Processor (STAP)

#### 3.1.1. Algorithm Architecture

**Figure 3:**
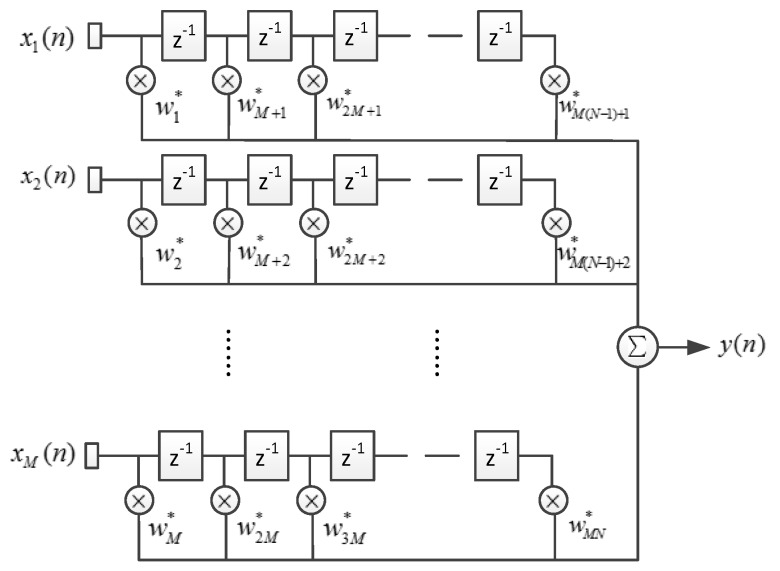
The architecture of a spatial-time adaptive processor (STAP).

A space-time adaptive processor (STAP) extends a space adaptive processor (SAP) by utilizing a finite impulse response (FIR) filter behind each element, thus introducing resolution in the temporal (frequency) domain and obtaining more degrees of freedom (DOF) [11]. Figure 3 shows the architecture of STAP, where M denotes the number of array elements, and N denotes the filter tap length. $x_{m}(n)$ denotes the complex sample data of element m at the time n, and y(n) denotes the complex output.

The input data vector x(n) and the weight vector w can be written as

(1) $$\begin{array}{l} x(n)=[x_{1}(n),x_{2}(n),\ldots ,x_{M}(n),x_{1}(n-1),x_{2}(n-1),\ldots ,x_{M}(n-1),\ldots ,\\ x_{1}(n-N+1),x_{2}(n-N+1),\ldots ,x_{M}(n-N+1){]}^{T} \end{array}$$

(2) $$w={\left[w_{1},w_{2},\ldots ,w_{MN}\right]}^{T}$$

Then y(n) can be written in the dot product form:

(3) $$y(n)=w^{H}x(n)$$

In GNSS anti-jamming applications, the weight vector is usually solved according to the linearly-constrained-minimum-variance (LCMV) criterion, in which case the optimized w can be written as [14]:

(4) $$w_{opt}=\frac{R^{-1}s}{s^{H}R^{-1}s}$$

where s is the constraint vector and R is the correlation matrix of the input data which is defined as follows:

(5) $$R=E\{xx^{H}\}$$

Assuming that the input data have a zero mean, then R is also the covariance matrix. In practice, it is usually estimated by

(6) $$\hat{R}=\frac{1}{K}\sum_{k=0}^{K-1}x(n-kN)x{\left(n-kN\right)}^{H}$$

where parameter K denotes the number of snapshots taken to estimate the covariance matrix. The parameter k is multiplied by N to ensure that the successive input data vectors do not overlap with each other, thus can be regarded as independent.

To implement adaptive beamforming, s can be set to a steering vector of one visible satellite which can be written as

(7) $$s={\left[0^{T},\ldots 0^{T},{s_{1}}^{T},0^{T},\ldots ,0^{T}\right]}^{T}$$

where 0 is the zero vector of M×1 and $s_{1}$ can be written as

(8) $$s_{1}={\left[e^{j\theta_{1}},e^{j\theta_{2}},e^{j\theta_{3}},\cdots e^{j\theta_{M}}\right]}^{T}$$

where $\theta_{i}(i=1,2,\cdots M)$ is the carrier phase shift of antenna element i due to the geometrical relationship between the antenna array and the satellite. In practice, the steering vector can be calculated by the direction of the satellite and the orientation of the antenna array measured by an Inertial Measurement Unit (IMU). It is necessary to note that, s can be written as the simple form because some non-ideal factors of the antenna and RF front-ends are ignored. These factors include the radio pattern’s anisotropy of each antenna element, inaccuracy of the array geometry, the mutual coupling effect, the finite ground-plane effect, mismatches between RF channels, different delays of cable lines, *etc.* [7,17,18].

In adaptive nulling, s is usually set to

(9) $$s={\left[0,0,\ldots ,0,1,0,0,\ldots ,0\right]}^{T}$$

in which the values are all zero except one. In this case, the approach is also called the power inversion (PI) method [14].

#### 3.1.2. Configurable Parameters

The antenna element number M and the filter tap length N are two basic parameters of STAP. In this project, M can vary from 4 to 8, while N can be equal to or less than 10. This makes that the size of R vary from 4 × 4 to 80 × 80. As we will see, this range of the covariance matrix scale will have an important influence on the GPU-based implementation.

The number of snapshots K is another important parameter. On one hand, it determines how accurate the estimation of R is. An inadequate estimation may result in a distorted antenna pattern far from the expected. Fante has stated in [15] that effective nulling can be achieved when K=4MN, which is a relatively small number. However, larger K surely helps to get a more accurate estimation of R in a stationary process. On the other hand, K decides the updating period of w together with N in our implementation. When N is fixed, the smaller K is, the more frequent the weight vector updates. Normally, we hope the weight vector has a short updating period in order to adapt to the fast changing environment. In summary, the choice of K is a balance between the estimating accuracy of R and the adapting capability. In this paper, K is set to vary from 100 to 1000, while larger K is possible but will not likely bring any obvious benefits.

Sometimes an inadequate estimation of the covariance matrix is inevitable, especially when the environment statistical characters are time-variant. One way to ameliorate the side effects brought by inadequate estimation of the covariance matrix is diagonal loading [19], by which we mean that $\hat{R}$ is added by a diagonal matrix as follows:

(10) $${\hat{R}}_{dl}=\hat{R}+\sigma_{dl}I$$

where $\sigma_{dl}$ is defined as the diagonal loading factor and I is the identity matrix. In [20] $\sigma_{dl}$ is set to be 3 or 10 dB greater than the thermal noise power. In this paper, $\sigma_{dl}$ can be configured to a fixed number, or more reasonably, can be set to be proportional to the trace of the covariance matrix, meaning that

(11) $$\sigma_{dl}=\alpha_{dl}\times \text{trace(}\hat{R})$$

where $\alpha_{dl}$ is a small number. In our experiments, it is also found that diagonal loading has a compensating effect when the interference is very strong, in which case ill-condition of the covariance matrix causes that the eigenvalues representing the noise are too small compared to the biggest eigenvalue, thus cannot be expressed precisely due to the precision of floating point numbers. The drawback of diagonal loading is that it can reduce the depth of nulls in the antenna pattern. In our implementation, the way how diagonal loading is conducted is configurable.

In both beamforming and nulling, selection of a reference tap is required. The reference tap corresponds to the non-zero elements in s. Moore has stated in [21] that reference tap selection has some kind of relationship with the output signal distortion. Simulation results also showed that selecting the center tap as the reference offers the best overall performance [21]. In spite of this, we leave the reference tap index as a parameter to be configured.

To conclude this subsection, the configurable parameters in STAP and their value ranges are summarized in Table 2.

### 3.2. Spatial-Frequency Adaptive Processor (SFAP)

#### 3.2.1. Algorithm Architecture

**Figure 4:**
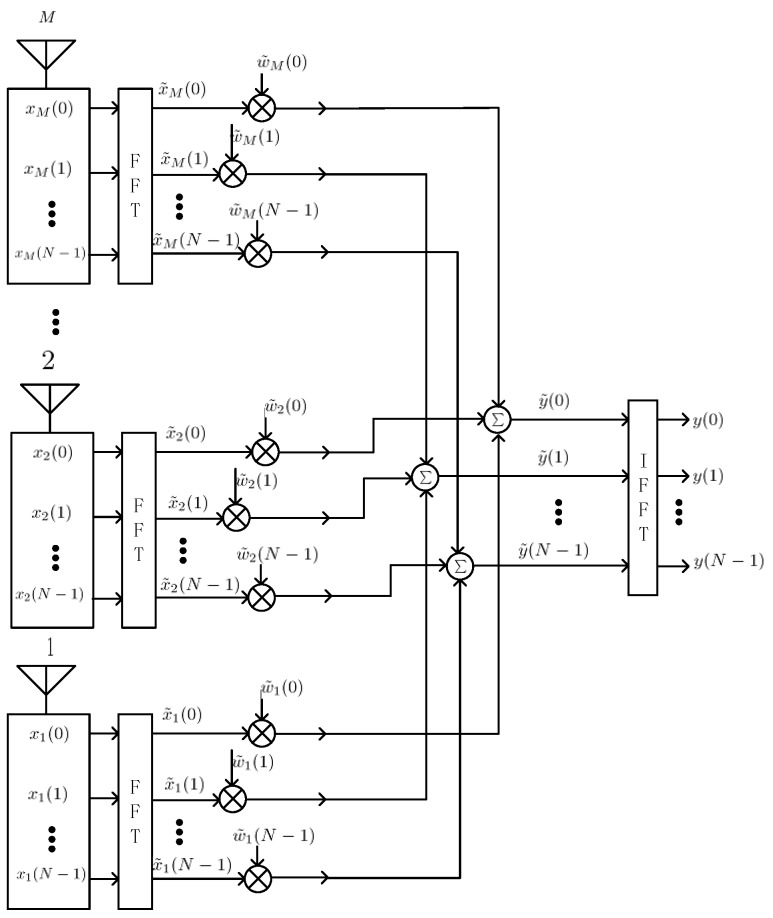
Architecture of the spatial-frequency adaptive processor (SFAP) with M antenna elements and a DFT length of N.

Spatial-frequency adaptive processor (SFAP) is proposed as an alternative of STAP to reduce the size of the covariance matrix to be inversed [16]. Figure 4 depicts the schematic overview of the spatial-frequency adaptive processor. Here, $x_{m}(n)$ (m=1,2⋯M, n=0,1,⋯N−1) denotes the complex sample data of element m at time, and ${\tilde{x}}_{m}(l)$ denotes the frequency sample of antenna m in frequency bin l after the discrete Fourier transform (DFT) module. In this paper, we use the same letter N to represent both the filter tap length in STAP and the DFT length in SFAP. The meaning should be obvious in the specific context.

In each frequency bin the output $\tilde{y}(l)$ is calculated by

(12) $$\tilde{y}(l)=\tilde{w}{\left(l\right)}^{H}\tilde{x}(l)$$

where $\tilde{w}(l)$ is the weight vector and $\tilde{x}(l)$ is the input vector, both in the frequency domain.

The weight vector is given by

(13) $$\tilde{w}(l)=\frac{\tilde{R}{\left(l\right)}^{-1}s}{s^{H}\tilde{R}{\left(l\right)}^{-1}s}$$

where $\tilde{R}(l)$ is the covariance matrix of frequency bin l which is defined as

(14) $$\tilde{R}(l)=E\{\tilde{x}(l)\tilde{x}{\left(l\right)}^{H}\}$$

In practice $\tilde{R}(l)$ is usually estimated by

(15) $$\hat{\tilde{R}}(l)=\sum_{i=0}^{K-1}\tilde{x}(l,i)\tilde{x}{\left(l,i\right)}^{H}$$

where $\tilde{x}(l,i)$ denotes the input vector in frequency bin l at snapshot i, and K is the snapshot number used to estimate $\tilde{R}(l)$.

In nulling, s can be set to

(16) $$s={\left[1,0,0,\cdots ,0\right]}^{T}$$

In beamforming, s is set to be the steering vector.

#### 3.2.2. Configurable Parameters

The three primary parameters of SFAP are M, N and K. For this study, M varies from 4 to 8, while N is set to be a power of 2 for the computational efficiency of FFT. In this project, N can be 1024 at maximum. The parameter K determines the weight updating period together with N, and influences the accuracy of the estimation of $\tilde{R}(l)$. Besides, diagonal loading is also used to desensitize the covariance matrix estimation, just like in the situation of STAP.

In addition to the four parameters above, Gupta has shown in [16] that when the interference scenario consists of narrow-band as well as wide-band sources, windowing the time domain samples leads to improved performance in SFAP. He also demonstrated that 20% to 25% sample overlapping is needed to achieve the optimum performance. Thus in this project, we leave the window type and the overlapping ratio as two configurable parameters.

To conclude this subsection, all the configurable parameters in SFAP are summarized in Table 3.

## 4. A Software-Defined Approach to the Anti-Jamming Platform

### 4.1. Software Architecture

The software is the part of the testbed that needs the most development efforts. In this project, we use Visual Studio 2010 (Microsoft, Redmond, WA, USA) and CUDA® 7.0 (NVidia, SantaClara, CA, USA) on the operating system of Windows 7 as the developing environment. Strictly speaking, Windows 7 is not regarded as a real-time operating system, but real-time applications developed on it are still possible due to the strong processing power of the processors. The software is developed in C++.

**Figure 5:**
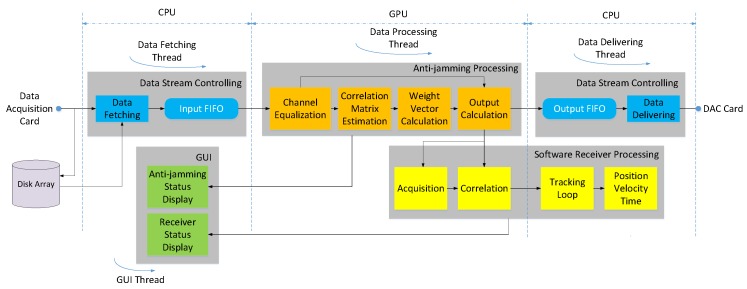
Software architecture.

Figure 5 shows the architecture of our software implementation. The software is mainly divided into four modules, namely the data stream controlling module, the anti-jamming processing module, the software receiver processing module and the GUI module, each of which can be further divided into several function units. These modules are driven by four threads, which are for data fetching, data delivering, data processing and GUI display, respectively. Some units are allocated to the GPU, while others are allocated to the CPU, just as shown in the figure. This allocation is mainly based on the data intensity of each task.

#### 4.1.1. Data Fetching and Delivering

In the data fetching thread, the data fetching unit receives raw data from the data acquisition card in packet form, and then send them to the input first-in-first-out (FIFO) buffer unit after the packet headers are parsed and stripped. The input FIFO buffer is allocated on CPU memory and aims to guarantees the continuity of the data flow. In the real-time mode, the raw data are extracted at the output end of the buffer by the data processing thread for following processing. The occupancy of the buffer should stay at a low level, which indicates that the data processing speed is faster than the data arriving speed. Instead of transferring the raw data to the input FIFO buffer, the data fetching unit can also log the raw data to the disk array directly for post-processing. Thus, in the post-process mode, the data fetching unit extracts the raw data from a file on the disk array.

If a standalone hardware receiver is used, then the data delivering thread is created to receive the output data from the anti-jamming processing module. The data are first transferred to the output FIFO buffer allocated in CPU memory, then extracted by the data delivering unit and packetized. Once an interrupt signal from the DAC card arises requiring for new data, a data packet is send to the card. Under normal conditions, occupancy of the output buffer should be neither full nor empty, and stay stable. The input and output FIFOs are the main contributions of the total latency of this system.

#### 4.1.2. Data Processing

The data processing thread is responsible for anti-jamming and software receiver processing. It has been well known that mismatches of the RF channels and different cable delays can reduce the interference mitigation performance seriously [23,24,25], so in this project, the multi-element raw data are first equalized by the channel equalization unit. The channel equalization is realized by filtering the raw data of each channel by a FIR filter. The filter coefficients are obtained beforehand through a calibration process, in which a strong white Gauss noise (WGN) signal is split into eight paths, each of which is injected to a RF channel through the LNA input. Then the output data of each ADC channel are used to train the coefficients using the Wiener filtering method. The calibration process may be conducted over time due to aging of the RF devices.

Besides channel equalization, covariance matrix estimation, weight vector calculation and output calculation are the three basic steps in both STAP and SFAP when the SMI method is used. These three units are the most computational intensive parts of the whole software, so must be carefully programmed and tuned. The implementation details will be introduced with emphasis in following subsections. For SFAP, a DFT unit and an IDFT unit should also be added, but they are ignored in Figure 5 for brevity.

If the integrated software receiver is used, then the output data after the interference suppression are transferred to the software receiver processing module for acquisition, tracking and position-velocity-time (PVT) calculation [26,27]. Of particular importance is that for beamforming, the input data to the receiver include multiple channels, each of which corresponds to a particular beam. In this project, both the common software receiver and the beamforming software receiver are modified versions of STARx, the implementation of which has been introduced very well in [6], thus is ignored in this paper.

### 4.2. GPU Architecture and Programming Model

The rest of this section focuses on the implementation of STAP and SFAP on the GPU. Firstly, it is necessary to give a brief review of the GPU architecture and its programming model.

#### 4.2.1. Thread Hierarchy

CUDA uses a single-program-multiple-data (SPMD) paradigm, which means that different data are processed by the same program concurrently. This is based on the hardware architecture of the GPU. In general, one single GPU contains a number of Streaming Multiprocessors (SMs), each of which is an array of Streaming Processors (SPs). Each SP is a fully pipelined, in-order microprocessor that executes a set of instructions serially, while many SPs in the same or different SMs can execute at the same time. For instance, GeForce GTX 980 which uses GM204 GPU contains 16 SMs, each of which contains 128 SPs, so that as many as 2048 threads can be executed at the same time.

In CUDA a kernel function executes a specific task, and generates a large number of threads to exploit data parallelism after launched. On programming logic level, these threads are further divided into blocks within the same grid. Each thread is distinguished from others by its block index in the grid (denoted by blockIdx) and thread index in the block (denoted by theadIdx). When mapping the programming logic architecture to the hardware architecture, each block must reside on one single SM, while each SM can contains more than one block at the same time. This determinates that the number of threads in one block must not exceed a certain value due to limited resources per SM, which include the number of SPs, the amount of shared memory as well as the register file size. However, the number of blocks within one grid can be very large, which is as much as 65,535 generally. Details about thread hierarchy in CUDA can be found in [28,29,30].

#### 4.2.2. Memory Hierarchy

Besides the execution model described above, data memory also plays an important role in GPU programming. Memory on the CPU side is called host memory in GPU programming context, whereas within the GPU, global memory, shared memory and registers are the main concerns. Global memory is off-chip and public to all threads within the grid. Accessing it is very slow compared to shared memory and registers. Shared memory resides on SM and are used as L1 cache. It is visible to all threads within the same block and can be accessed much faster than global memory, but the amount of it is limited (generally 48 KB per SM). Registers have the smallest accessible domain which is restricted to within one single thread, but they have the smallest access latency that is ignorable. The register file size is limited, and often it is the complier’s role to decide how much of it is used.

It is well known that data transfer between global memory on GPU and host memory on CPU is very costly, so should be minimized as much as possible. Besides, it is always preferable to let the global accesses to coalesce, which is perhaps the most important performance consideration in CUDA programming [31].

Another key point of memory optimization is to use as much fast-access memory and as little slow-access memory as possible to maximize bandwidth [10]. Caching the frequently-used data to shared memory and registers can significantly lower memory access cost. However the amount of them is limited, thus they should not be overused. Details about memory in GPU programming can also be found in textbooks and tutorials such as [28,29,30].

#### 4.2.3. The Occupancy Metric

Memory access is often the bottleneck of performance, so it is important to launch as many active threads onto the hardware as possible to keep the SMs busy and hide latencies through context-switching. Occupancy is a metric to measure how busy the hardware is, and is defined as the ratio of the number of actual active threads to the maximum number of possible [31]. It is determined by the kernel function and the resource offered by the SM, which includes the amount of registers and shared memory, as well as the maximum thread number per SM. Low occupancy can result in performance degradation in most cases, so programmers are encouraged to prevent this from happening. In order to reach this goal, the block size, the used registers per thread, as well as the used shared memory per block should be reduced. However, Volkov has showed in [32,33] that under certain circumstances, faster codes may run at a lower occupancy. The key point is to use more registers and do more parallel work per thread.

### 4.3. Batched STAP Implementation

This subsection introduces the implementation of STAP, in which a batched method is used to make the best use of the GPU parallelism.

The data going to the anti-jamming processing module are in a stream form. To reduce the data transfer count as well as to take advantage of the GPU parallelism, the data stream is divided into chunks in host memory, then the whole data in one chunk are transferred onto global memory at a time. In this project, the time length of one chunk is 50 ms. This is the result of a balance between data transferring time and memory cost. Thus, the sampling period number within one chunk is

(17) $$L_{\text{chunk}}=20\text{ MSPS }\times 50\text{ ms }=10^{6}\text{ samples}$$

**Figure 6:**
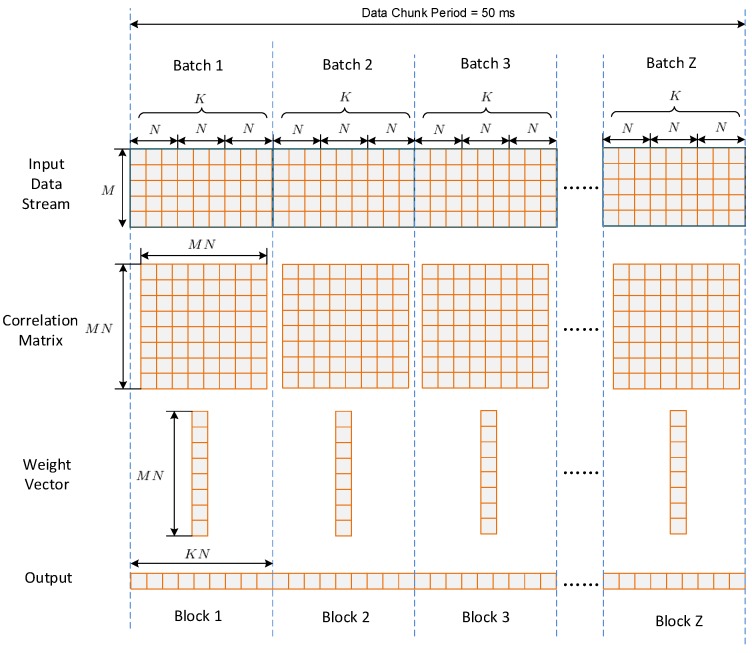
The batched implementation of STAP.

Figure 6 depicts the batched implementation of STAP. After one chunk of input data has been transferred onto global memory, the chunk is further divided into Z batches, while each batch contains K non-overlap input vectors of MN×1. Thus, Z can be calculated by

(18) $$Z=\text{ceil}(\frac{L_{\text{chunk}}}{NK})$$

where “ceil” denotes the function of rounding up. In the input data stream shown in Figure 6, rows corresponds to different antenna elements, while columns corresponds to different sampling period. For each batch, one covariance matrix of MN×MN is estimated, and one weight vector of MN×1 is calculated using the SMI method. Then KN outputs are calculated from the input data using the same weight vector. Thus, each batch corresponds to a weight updating period, which is calculated by

(19) $$T_{update}=KNT_{s}$$

where $T_{s}$ is the sampling period and N is the filter tap length.

It can be seen that each batch can be regarded as a small “problem” independent with others. One way to calculate these problems is to process them one by one. However, this is usually inefficient since the scale of each problem is not big enough to obtain a high occupancy. Furthermore, the kernel function invoking expense is costly when the batch number per chunk is large. In order to address this issue, a batched method is proposed in this paper, in which all the batches within one chunk are processed concurrently by a single kernel launch.

In the rest of this subsection, the implementation of the first two calculation steps in STAP are introduced in detail, namely the covariance matrix estimation and the weight vector calculation, respectively. The output calculation is implemented by a kernel function which is called *gpu_output_batched*. This calculation step contains mainly matrix-vector multiplications, thus the implementation details are ignored here.

#### 4.3.1. Batched Covariance Matrix Estimation

The covariance matrix estimation is one of the computational hotspots and should be fully optimized to meet the real-time requirement. In practice, the covariance matrix is estimated by a time average method given by Equation (6). As an alternative, this equation can be rewritten in matrix form as follows.

(20) $$\hat{R}=\frac{1}{K}XX^{H}$$

where X is the input data matrix written by

(21) $$X=[x^{T}(n),x^{T}(n-N),\ldots ,x^{T}(n-KN+N)]$$

A specialized kernel function called *gpu_Rxx_batched* is developed to calculate the Z correlation matrices within one chunk concurrently. In the kernel, each covariance matrix of one batch is mapped to a single block, while each element to be calculate in the covariance matrix is mapped to a single thread within the block. Only elements on and below the diagonal are calculated for the conjugate symmetry of the covariance matrix.

**Figure 7:**
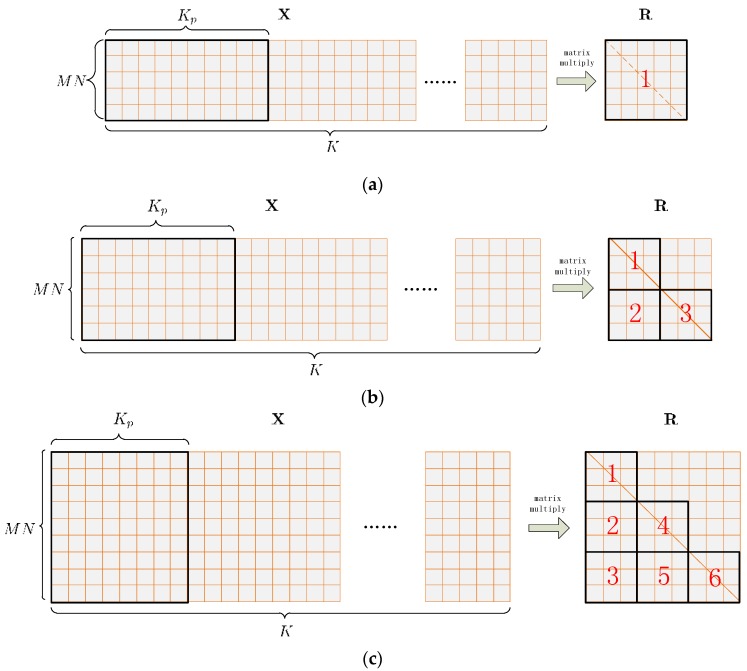
Schematic of the covariance matrix estimation. (**a**) Covariance matrix estimation of size smaller than 30 × 30; (**b**) Covariance matrix estimation of size from 30 × 30 to 59 × 59; (**c**) Covariance matrix estimation of size from 60 × 60 to 80 × 80.

Each element in the input data matrix is used many times, so is cached onto shared memory to eliminate reluctant accesses to global memory. This also helps the global memory accesses to coalesce. However, special attention should be paid not to overuse shared memory, because the amount of it is limited. For GTX 770, the amount of shared memory is 48 kB per SM at maximum. This results that when MN and K are relatively large, the size of the input data matrix can be quite big, which makes caching the whole of it onto shared memory within one single transfer inappropriate or impossible. To address this issue, the input data matrix is further divided into multiple successive slices, as shown in Figure 7 on the left side. Each slice contains $K_{p}$ input vectors, and is cached and processed one after another. The parameter $K_{p}$ needs to be carefully tuned to obtain the best performance.

Each element of the covariance matrix is stored in the on-chip register file while being calculated by the corresponding thread. In this project the size of the covariance matrix varies from 4 × 4 to 80 × 80, so *gpu_Rxx_batched* should adapt all the various sizes. On the other hand, the thread number per block is limited. For GTX 770, the maximum thread number per block is 1024. For a covariance matrix of 80 × 80, if one thread calculates only one element on or below the matrix diagonal, then there needs to be 3240 threads per block, which exceeds 1024. To address this issue, the covariance matrix is portioned into tiles, as shown in Figure 7 on the right side. For a covariance matrix whose size is smaller than 30 × 30, one tile occupies the entire matrix. For a covariance matrix whose size varies from 30 × 30 to 59 × 59, three tiles cover all the elements on and below the diagonal. For a covariance matrix whose size varies from 60 × 60 to 80 × 80, the number of tiles increases to 6. For each covariance matrix, the tiles are processed serially by the block, which means that if the covariance matrix is divided into v tiles of t×t, then each block should contains t×t threads and each thread should calculate v matrix elements. Table 4 shows the block size corresponding to different covariance matrix sizes. Thus, the number of threads per block is decreased to the size of a tile, which is less than 901, as shown in Table 4. At last, the resulted covariance matrix in each batch is copied to global memory.

#### 4.3.2. Batched Cholesky Solving

The calculation of the weight vector can be viewed as a problem of solving linear equations, such avoiding the computational cost of matrix inversion. This solving process can be divided into three steps. Firstly, the covariance matrix can be factorized by the Cholesky method into a multiplication form which is given by

(22) $$R=LL^{H}$$

where L is a lower triangular matrix. Then the vector $b=R^{-1}s$ is obtained by two iteration processes, which are Ld=s and $L^{H}b=d$ in turn. Finally, b is normalized to obtain the desired weight vector, which is

(23) $$w=\frac{b}{s^{H}b}$$

**Figure 8:**
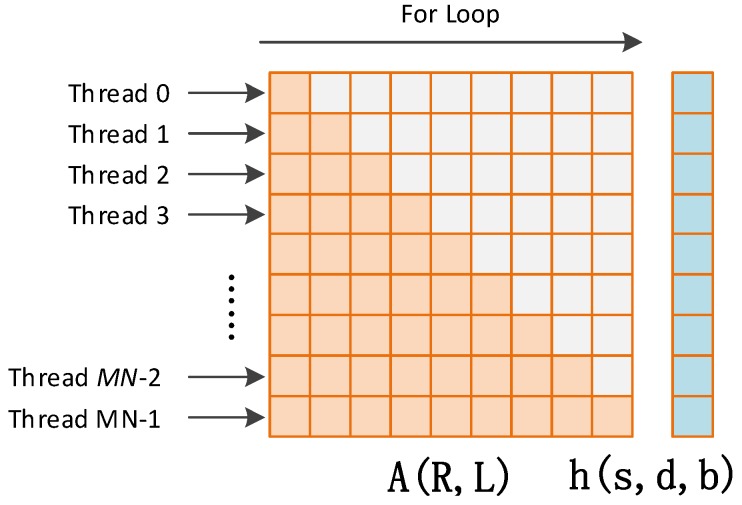
Architecture of the Cholesky solver.

Parallel implementation of Cholesky solver has been investigated in previous works. In [9] Molero proposed a batched Cholesky solver for local RX anomaly detection in hyper spectral images. The method calculates hundreds of different and independent small problems simultaneously, such reaching a high degree of parallelism on a GPU. In this work we adopt a similar implementation method by developing a kernel called *gpu_cholesky_batched*. In this kernel, each block processes one problem and contains MN threads, while each thread corresponds to one row of the covariance matrix, as shown in Figure 8. The covariance matrix and the constraint vector are cached onto shared memory at the beginning of the kernel launch. The algorithm of the Cholesky solver for each block is as shown in Algorithm 1 in Appendix A.

From Figure 8 and Algorithm 1, it can be seen that the covariance matrix R and the lower triangle matrix L share the same memory in the Cholesky factorization. The same is for vector s, d, and b in the forward and backward iterations. Thus, the used shared memory is reduced. It can also be seen that there are two “for” loops in each block, and every iteration in the loop depends on results of the former iteration. This means that the operations in each block are not fully parallelized. This is also why the costly function *syncthreads* which acts as a barrier where all threads within one block must wait before any is allowed to proceed is repeatedly used. However, this lack of parallelism is balanced by the fact that all the batched are processed in parallel.

### 4.4. Batched SFAP Implementation

Similar to STAP, one chunk of data is also divided into batches. However, one difference in the batched implementation of SFAP with STAP presented in this paper is that, since the key point of SFAP is to do SAP in each frequency bin, DFT and IDFT should be carried out at the beginning and end of the whole process, respectively. Thus, after DFT, each batch of data is further divided into N small batches corresponding to N frequency bins. Here, N denotes the DFT length. The DFT and IDFT on a GPU can be done by the off-the-shelf library CUFFT® which is well tuned, so we use functions in it directly instead of reinventing the wheel.

**Figure 9:**
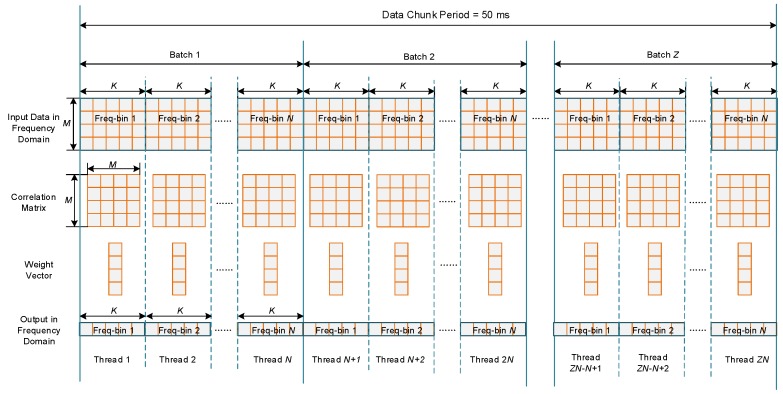
Data layout in the spatial-frequency adaptive processor (SFAP) after the FFT.

The batched implementation of SFAP is depicted in Figure 9. For brevity, only calculation steps in frequency domain are showed. For each frequency bin of each batch, one covariance matrix estimate is calculated, then one weight vector is generated. After that, outputs in frequency domain are calculated, ready to be transformed back to time domain by IDFT. The data of each batch in time domain may overlap with adjacent batches at the two ends, so the weight updating period is calculated by

(24) $$T_{update}=(1-overlap)KNT_{s}$$

where overlap is the overlapping ratio.

The key point in designing the kernels *gpu_Rxx_batched* and *gpu_cholesky_batched* in STAP is that they should adapt to the different sizes of the covariance matrices, which vary from 4 × 4 to 80 × 80. Different from this, the covariance matrix size in frequency domain is relatively small, which varies from 4 × 4 to 8 × 8. This makes it possible to further improve the performance by certain measures.

#### 4.4.1. Kernel Function Design Strategy

It is generally recommend that in order to hide latencies, one should run more threads per multiprocessor [10], which means that a high occupancy is preferred. However, Volkov has stated in [32,33,34] that it is possible to gain high speed up at low occupancy. The key is to calculate more outputs per thread and hide latency by instruction-level optimization (ILP). Our batched implementation of SFAP is based on this strategy.

The kernel we developed is called *gpu_Rxx_cholesky_weight_output_sfap*, which indicates that all the calculation steps in frequency domain are completed in one single kernel function. In this kernel, each small batch (*i.e.*, one frequency bin of one batch) is assigned to a single thread. The covariance matrix and the constraint vector s are allocated as registers instead of shared memory. These registers are reused as much as possible to avoid more register allocation, thus more threads can be loaded to SM at the same time. Registers are further saved by only loading and calculating the matrix elements on and below the diagonal. The process of the kernel is as follows: firstly, the input data of one small batch are loaded to the register file by one thread, then multiplied and accumulated to estimate the covariance matrix, just as depicted in Equation (15). Secondly, Cholesky factorization is conducted and the weight vector is generated after the two-phase iteration. All the operations are strictly serial within one thread, so the costly function *syncthreads* does not need to be called repeatedly. Finally, the input data are read from global memory again to be weighted by the weight vector and summed on registers, and then written back to global memory.

Performance can benefit from at least two aspects. Firstly, all the three calculation steps are integrated into one single kernel, reducing both accessing to global memory repeatedly and the multiple kernel launch overhead. Secondly, in memory hierarchy of GPU, registers are nearest to arithmetic logical units (ALUs) so have the minimum latency. Furthermore, it does not have the problem of bank conflicts and thread synchronization which shared memory suffers from [30]. Although the input data reading operation from global memory is time costly, but once it is completed, following operations can be accomplished on-chip with nearly no memory latency.

To improve latency within one thread, IPL is used, which means overlapping independent operations so that one can run while the others are waiting for memory accesses to be finished [33]. Besides, loops are unrolled to gain better performance [34]. Since CUDA does not support dynamic allocation of registers, five versions of the kernel are developed, respectively, for 4 to 8 antenna elements.

#### 4.4.2. Performance Preview

**Figure 10:**
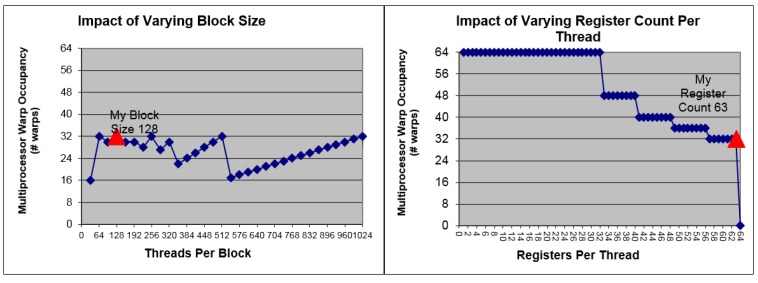
Results of occupancy calculations. (**Left**) Impact of varying block size on occupancy; (**Right**) Impact of varying register count per thread on occupancy.

In order to get a deeper insight into the problem, we use the GPU occupancy calculator provided by NVIDIA to give a performance preview [35]. The kernel tested is the 8-elements version which has the highest computational quantity. In the calculator, the Compute Capability (CC) is set to 3.0, which is the same with GTX 770, and block size is set to 128. Results are presented in Figure 10. It can be seen that 63 registers are allocated by the complier per thread, which is the maximum number of available registers in a GPU of CC 3.0. This results in that 32 warps are active per SM. In a GPU every 32 threads are bound to be a warp, so the total number of active threads per SM is 1024. The maximum warp number is 64 per SM, so the occupancy we get is 50%, which is low. This is due to that the allocated registers per thread are too many, which can be seen in the right graph presented in Figure 10. However, we will show in the next section that a considerably good performance can still be obtained.

### 4.5. Precision Consideration

Precision is an important factor influencing the anti-jamming performance in both signal digitalizing and calculation. In this platform, the ADC has a resolution of 14 bits, resulting in an effective dynamic range of more than 65 dB which is needed to avoid saturation in the digital domain while sampling the received signal containing interference [36]. A 14-bit resolution is also considered to be enough to limit the side effect on interference cancellation brought by quantization errors [37].

After being digitalized, the sampled raw data are transferred to processors for calculation. In a hardware implementation using a FPGA, data are usually processed in a fixed point form, and the precision usually depends on the truncated bits after multiplication or other operations. However, in a general-purpose processor like CPU and GPU, data are usually manipulated in floating point type to get higher precision, because fixed point operations will not bring extra benefit in general. In particular for GPU, 32-bit single-precision operations are much faster than 64-bit double precision operations. For example, on a GPU of Compute Capability 3.0, the number of single-precision operations per clock cycle per SM is 192, while that of double precision operations is only 8 [30]. Thus, the fixed point raw data are transformed to single-precision type before being calculated by kernel functions.

It is known that the relative precision of 32-bit single-precision operations is about 1 × 10^−7^ [38]. This implies an upper limit of the eliminable jamming power, which corresponds to the situation when the ill-condition degree of the covariance matrix is so large that the minimum eigenvalue cannot be represented with enough precision. In practice, this effect can be compensated by diagonal loading.

In situations when accuracy is of lower priority than the performance, the fast math library can be used instead of the standard math library in CUDA for further optimization. This is done by switching on the *–use_fast_math* compiler option. Test results show that using this method, the time cost of the *gpu_cholesky_batched* kernel can be reduced by about 15% to 25% according to different problem scales. In our implementation, this optimization option is adopted, and experiment results show that the anti-jamming performance can be maintained.

### 4.6. Whole Picture of the Testbed

**Figure 11:**
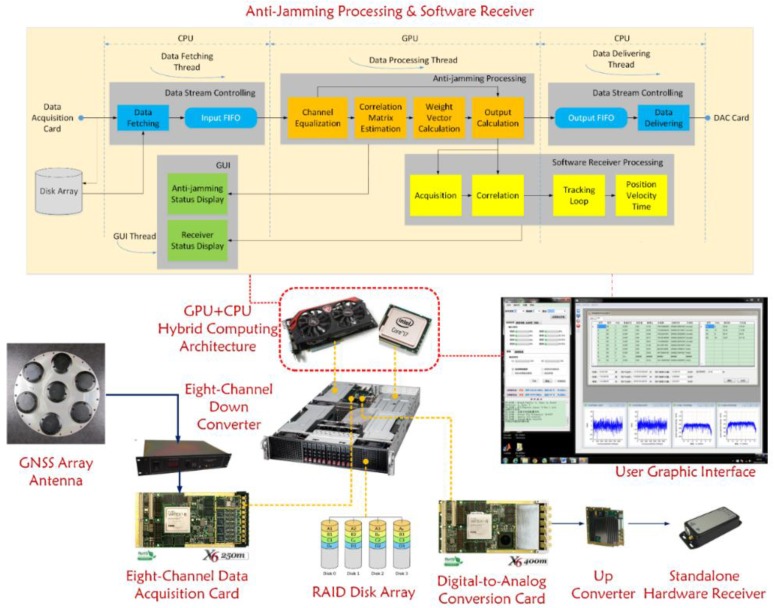
The complete schematic picture of the real-time testbed for adaptive GNSS array anti-jamming.

Until now, both the hardware and software of this testbed have been introduced in detail, so now it is time to give a whole picture of the platform, which is shown in Figure 11 below.

## 5. Computational Performance

The focus of the software-defined approach in the last section is to create programs efficient enough to meet the real-time requirement. In this section, the computational performance of both the STAP and SFAP implementation is measured by inserting CPU clock timers to the codes and measuring time costs of different calculation steps. It will be seen that, with our programming techniques, the real-time requirement can be satisfied very well for a wide range of the configurable parameters.

All the results in this section are obtained using GTX 770, which is a not so advanced GPU compared to the newly released GTX 980 or GTX TITAN X. It can be expected that better computational performance can be gained with more advanced GPU without modifying the program, so results in this section are conservative.

### 5.1. Computational Performance of STAP

**Figure 12:**
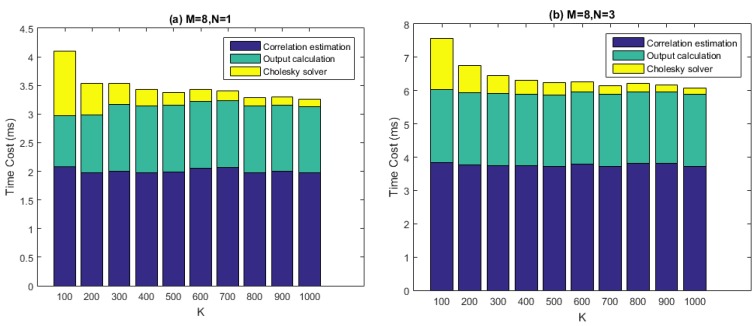

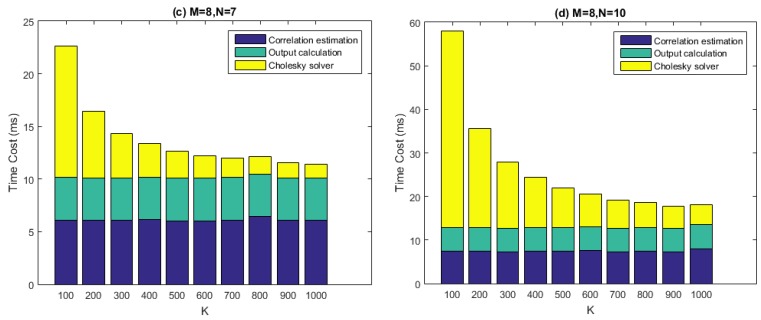
Performance of the batched STAP implementation. M denotes the number of antenna elements and N denotes the filter tap length. The real-time threshold is 50 ms. (**a**) Results of time cost when M is 8 and N is 1; (**b**) Results of time cost when M is 8 and N is 3; (**c**) Results of time cost when M is 8 and N is 7; (**d**) Results of time cost when M is 8 and N is 10.

Figure 12 depicts the computational performance measurement results of our batched STAP implementation. In the test, time costs of the three calculation steps, namely the covariance matrix estimation, the Cholesky solving, and the output calculation are measured for one chunk of input data, which is 50 ms long. The number of antenna elements (*i.e.*, M) is set to 8, which is the maximum in this project and represents the situation with the highest computational quantity. The filter tap length (*i.e.*, N) is set to 1, 3, 7, 10 respectively, while the number of snapshots taken to estimate the covariance matrix (*i.e.*, K) ranges from 100 to 1000 with a step size of 100. According to the last section, the number of batches (*i.e.*, the number of blocks, or grid size) is determined by N and K together. Block size of all the three kernels, which are *gpu_Rxx_batched*, *gpu_cholesky_batched*, and *gpu_output_batched*, is set to 256 as a result of carefully tuning.

From the figure it can be seen that, given particular values of M and N, the time costs of the covariance matrix estimation and the output calculation stay roughly consistent with K varying. This result makes sense because the arithmetic operations in the corresponding two kernels are relatively simple, thus global memory access may become the bottleneck of the performance. However, the time costs of Cholesky solving decreased with K increasing. This is because smaller K corresponds to more batches to be processed. The comparison between the four graphs also makes clear that the time costs of the Cholesky solving increase with N much faster than the other two calculation steps, which is partly because for *gpu_cholesky_batched*, arithmetic operations play the central role of performance, rather than memory accesses.

The real-time requirement demands that, given a data chunk of 50 ms, we must process it within 50 ms. This is achieved for most cases in Figure 12, except for the situation when M=8, N=10 and K=100. However, this set of parameters is not practical, since the relation K≥4MN is generally required to be satisfied for effectively interference mitigation. Thus, we can say that the real-time requirement is satisfied in a practical sense.

### 5.2. Computational Performance of SFAP

**Figure 13:**
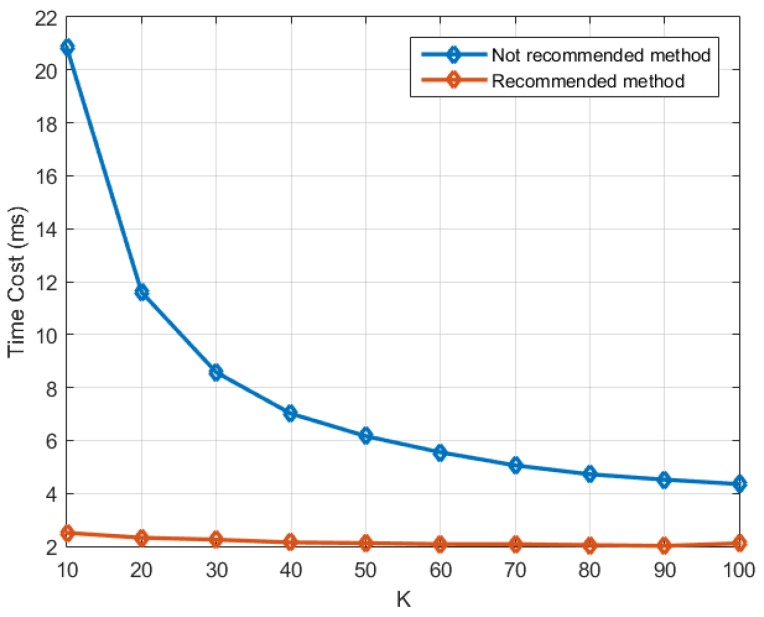
Comparison between the two implementation methods introduced in Section 4.3 and Section 4.4 in the context of SFAP. The parameters M and N were 4 and 256, respectively.

Before exploring the performance of the whole process, we compare the two implementation methods introduced in Section 4.3 and Section 4.4 respectively in the context of SFAP. In the recommended method introduced in Section 4.4, all the three calculation steps in the frequency domain are accomplished by the kernel *gpu_Rxx_cholesky_weight_output_sfap*, while in the not recommended method which is introduced in Section 4.3, they are accomplished by *gpu_Rxx_batched*, *gpu_cholesky_batched*, and *gpu_output_batched* respectively. In the test, one chunk of input data is used, which is 50 ms long. The number of antenna elements (*i.e.*, M) is set to 8, the DFT length (*i.e.*, N) is set to 256, and the parameter overlap is set to 0. K ranges from 10 to 100 with a step size of 10. Only time costs of the calculation in the frequency domain are measured, meaning that DFT and IDFT are not included. Figure 13 depicts the result, from which it can be seen that the recommended method outperforms the not recommended method a lot, and the smaller K is, the more obvious the improvement is.

**Figure 14:**
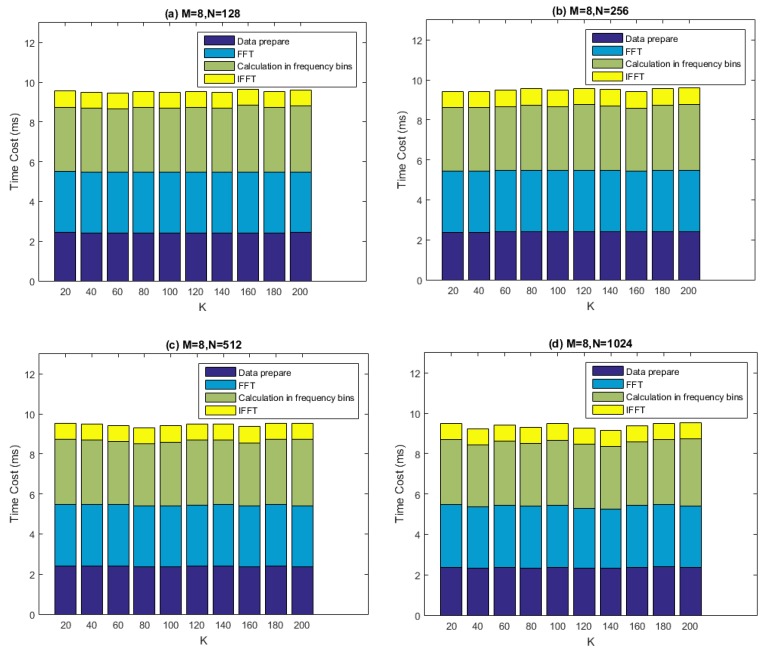
Performance of the batched SFAP implementation. M denotes the number of antenna elements and N denotes the FFT length. The real-time threshold is 50 ms. (**a**) Results of time cost when M is 8 and N is 128; (**b**) Results of time cost when M is 8 and N is 256; (**c**) Results of time cost when M is 8 and N is 512; (**d**) Results of time cost when M is 8 and N is 1024.

Figure 14 depicts the computational performance measurement results of our batched SFAP implementation using the recommended method. Here, M is set to 8, while N is set to 128, 256, 512 and 1024 respectively. The parameter K ranges from 20 to 200 with a step size of 20. The parameter overlap is set to 0.4, which is a conservative value. In the figure, the “data preparation” denotes the data arrangement before the DFT. It can be seen that the two most computationally costly parts of the whole process is FFT and the calculation in frequency domain (*i.e.*, *gpu_Rxx_cholesky_weight_output_sfap*). The total time cost per data chunk is less than 10 ms, leaving more than 40 ms for other operations, meaning that the real-time requirement is satisfied very well.

### 5.3. Real-Time Processing Time Distribution

The total time cost of the software includes not only the anti-jamming processing on the GPU mentioned above, but also the data transfer between device and host, as well as the software receiver processing. In this subsection we measure the time cost distribution among the three steps.

**Figure 15:**
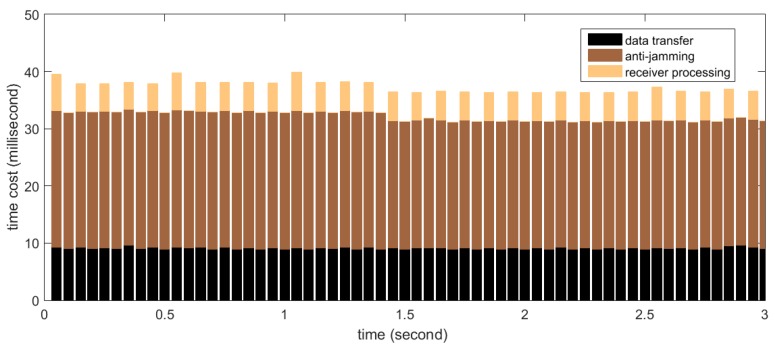
Time cost distribution for real-time processing with the space-time adaptive processor (STAP).

Firstly the real-time processing time distribution for STAP is measured. In the test, the number of antenna elements (*i.e.*, M) is set to 8 and the filter tap length (*i.e.*, N) is set to 10, thus the size of the covariance matrix is 80 × 80, which is the maximum in our implementation. The number of snapshots taken to estimate the covariance matrix (*i.e.*, K) is set to 500, resulting in the weight updating period to be 0.25 ms according to Equation (19). According to Section 5.1, this set of parameters represents computational complexity near to the maximum required by this project. The measurement results are shown in Figure 15. The *X*-axis in the graph corresponds to continuous time, segmented into processing periods of 50 ms length. The *Y*-axis corresponds to the time cost of each processing period. Shown as stacked bars are the data transfer (black), anti-jamming processing (dark brown) and software receiver processing (light brown). The software receiver processes data every 100 ms, so the bar representing the time cost of the receiver processing appears every two periods. It can be seen that for this set of parameters, more than 15 ms of idle time on average are left for operations other than the three kinds in the figure, so the real-time requirement is satisfied very well.

**Figure 16:**
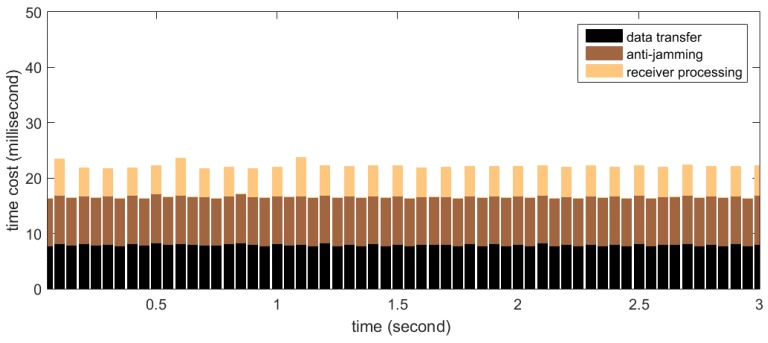
Time cost distribution for real-time processing with the space-frequency adaptive processor (SFAP).

The real-time processing time distribution for SFAP is measured in a similar manner with STAP. The number of antenna elements (*i.e.*, M) is set to 8 and the DFT length (*i.e.*, N) is set to 256. The number of snapshots taken to estimate the covariance matrix (*i.e.*, K) is set to 200 and the parameter overlap is set to 0.4, so the weight updating period is 0.768 ms according to Equation (24). According to Section 5.2, this set of parameters is representative, for the computational complexity of SFAP is almost constant with varying M, N and K. The results are presented in Figure 16. It can be seen that about 30 ms of idle time on average are left for other operations per 50-millisecond-long period, meaning that the real-time requirement is satisfied very well. Besides, the data transfer time is slightly less than STAP. This is due to that the data type of the input data in SFAP is INT16 whose size is 2 Bytes, while the data type of the input data in STAP is single precision floating point, whose size is 4 Bytes, resulting in that the total amount of the input data to be transferred to the global memory in SFAP is less than that in STAP.

## 6. Anti-Jamming Experiments

A major advantage of the platform is that the intermediate states of both the anti-jamming processing and the software receiver processing can be observed in the field or analyzed afterwards. These intermediate states include eigenvalues of the covariance matrix, updating weight vectors, C/N0 of each tracking channel, *etc*. More insights to the anti-jamming performance can be obtained based on these states. In this section, examples of adaptive nulling both in STAP and SFAP are given, showing how anti-jamming algorithms with different parameters react to the changing of the interference environment. Besides, the performance of adaptive beamforming is measured. Experiments for adaptive nulling are conducted using the BDS B3 signal, whose bandwidth is 20 MHz, while the experiment for beamforming is conducted using the GPS L1 C/A signal.

### 6.1. Jamming Scenarios

**Figure 17:**
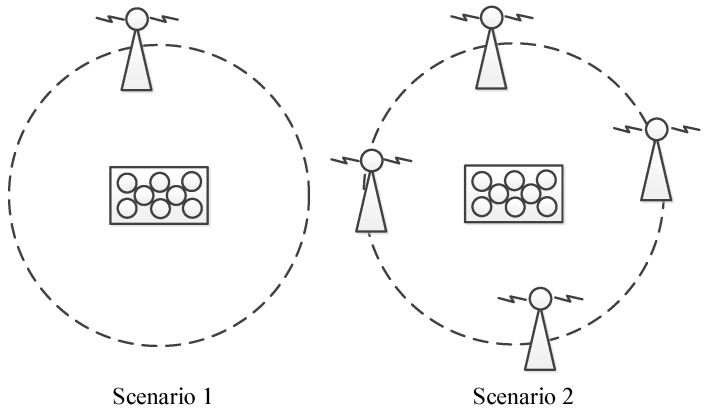
The two jamming scenarios used in the following experiments.

**Figure 18:**
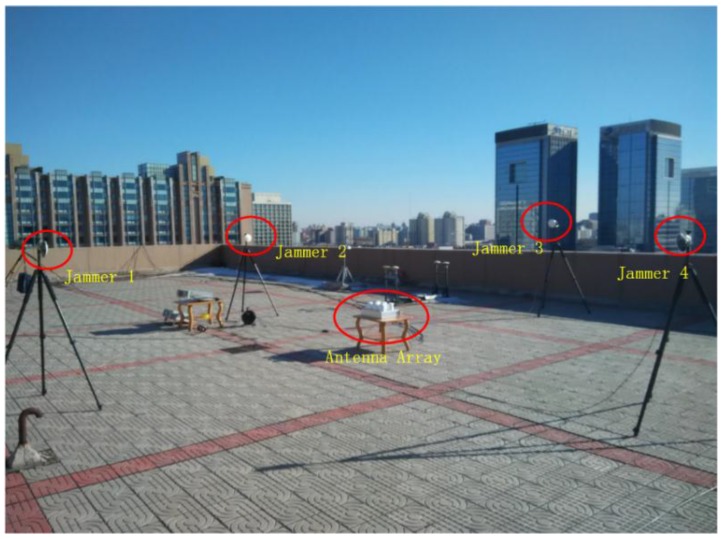
A photo of the experiment setup.

In this section, the adaptive array anti-jamming performance is measured in two different jamming scenarios, as shown in Figure 17. In the first scenario, there is one wideband jammer in the north direction of the antenna array, covering all the bandwidth of the BDS B3 signal which is 20 MHz. The jammer stays silent at the beginning to let the receiver capture signals from visible satellites and is turned on at about second 66 with an interference-signal-ratio (ISR) being about 60 dB. Then every more than 10 s, the jammer is strengthened gradually by 6 dB, increasing the ISR range from 60 dB to more than 90 dB. This scenario lasts for about 180 s. In the second scenario, four wideband jammers are located at the four ends of a square. All jammers stay silent at first, and then are turned on one by one, with an ISR of each jammer being about 45 dB. Information of the 4 jammers is presented in Table 5. This scenario lasts for about 100 s. Figure 18 is a photo of the experiment setup.

In order to compare the anti-jamming performance of different algorithms and parameters against the same interference environment, instead of running the testbed in a real-time mode, the multi-element signals received in the two jamming scenarios are digitalized and logged to the disk array respectively, after which the logged file on the disk array are processed in a post-processing mode. The results obtained in this way are the same with the real-time mode.

### 6.2. STAP Nulling Experiments

**Figure 19:**
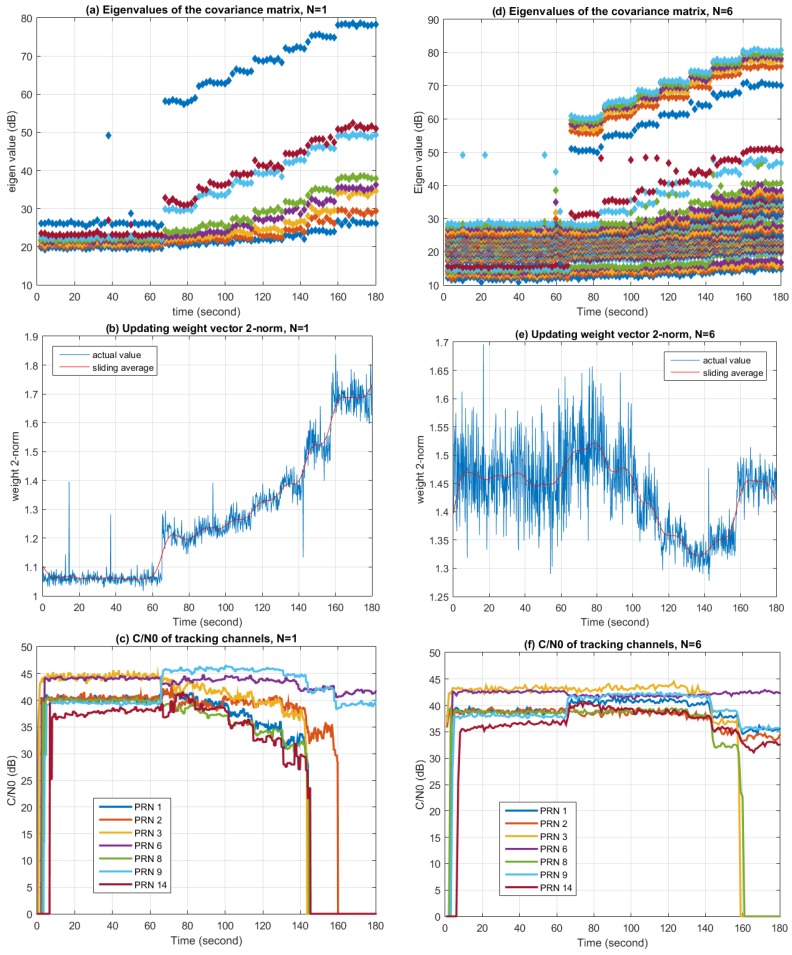
Performance of the STAP nulling during Jamming Scenario 1. (**a**) Eigenvalues of the corvariance matrix when N is 1; (**b**) 2-norm of the updating weight vector when N is 1; (**c**) C/N0 of the tracking channels when N is 1; (**d**) Eigenvalues of the corvariance matrix when N is 6; (**e**) 2-norm of the updating weight vector when N is 6; (**f**) C/N0 of the tracking channels when N is 6.

Firstly, the STAP nulling performance in Scenario 1 was measured. In the experiment, the antenna element number (*i.e.*, M) was set to 8, while the number of snapshots taken to estimate the covariance matrix (*i.e.*, K) was set to 200. In order to desensitize the side effect brought by inadequate estimation of the covariance matrix, diagonal loading was conducted according to Equations (10) and (11), where $\alpha_{dl}$ was set to 1 × 10^−6^. Figure 19 shows the changes of covariance matrix eigenvalues, the Eucidian 2-norm of the updating weight vector, and C/N0 with time, respectively. The left three graphs correspond to the situation when N=1. In this case, the STAP algorithm degrades to SAP, while the weight updating period is 10 microseconds. The right three graphs correspond to the situation when N=6, thus the weight updating period is 60 microseconds.

From Figure 19a,d, it can be seen that the eigenvalues increase with the jamming signal power. When N=1, the biggest eigenvalue is a lot larger than the second biggest eigenvalue, while the biggest five eigenvalues are close to each other when N=6. This indicates that while STAP provides more degrees of freedom than SAP, more degrees of freedom are also consumed in STAP by the wideband interference. In the ideal case where one wideband jammer exits in the environment, the largest eigenvalue in SAP represents the jamming signal, while other eigenvalues represent the noise and are more or less the same [39]. However, it can be seen that several large eigenvalues also appears in the actual experiment. This may be due to the antenna element mutual coupling effect and RF channel mismatches. It is also worth noting that the smallest eigenvalue increases with the jamming signal power because of the diagonal loading operation.

Figure 19b,e depict the changes of the Euclidian 2-norm of the updating weight vector. Although this metric does not have an obvious physical meaning, it provides a simple way to indicate the value changes of the weight vector along with the environment. In the case of N=1, the norm increases with the jamming signal power, while in the case of N=6, the norm changes with the environment, but the relation is not intuitive. This result also confirms that by observing the value or other characteristics of the weight vector, changes in the interference environment can be observed.

Figure 19c,f depict the C/N0 changes of several tracking channels during processing. Since the jammer is very strong, if no anti-jamming algorithms are used, all the tracking channels will lose tracking (*i.e.*, C/N0 = 0) immediately after the jammer is turned on. But with the help of the anti-jamming algorithms, channels can still be tracked, as presented in the graphs. It can be seen that when the jammer is turned on, there is a sudden change in C/N0 for most tracking channels, while some of them increase and some of them decrease. This is due to the sudden distortion of the antenna array radio pattern, resulting changes of the gain in different directions, since in the PI method the array pattern toward the satellites are not constrained. In the case of N=1, after the jammer is turned on, C/N0 of each tracking channel decrease with the increasing jamming power, and finally lose tracking when the jamming power exceeds a certain threshold. In the case of N=6, the trend of the change is similar, but the jamming power threshold after which C/N0 begins to decrease is higher than the case of N=1, and in the end more tracking channels remain locked. This proves that more filter taps in STAP can lead to better performance in interference similar to Scenario 1.

**Figure 20:**
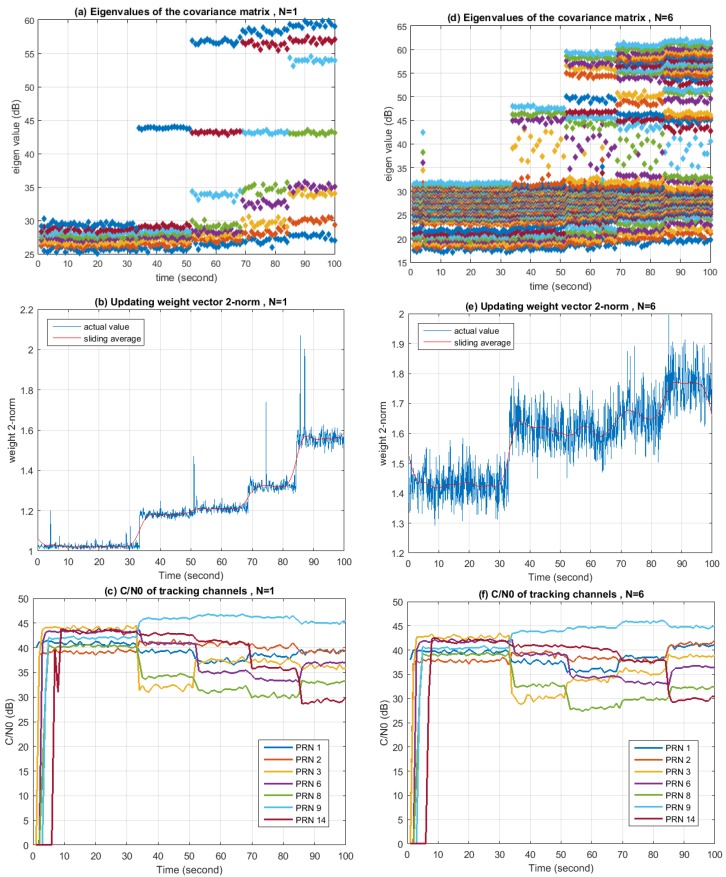
Performance of the STAP nulling during Jamming Scenario 2. (**a**) Eigenvalues of the corvariance matrix when N is 1; (**b**) 2-norm of the updating weight vector when N is 1; (**c**) C/N0 of the tracking channels when N is 1; (**d**) Eigenvalues of the corvariance matrix when N is 6; (**e**) 2-norm of the updating weight vector when N is 6; (**f**) C/N0 of the tracking channels when N is 6.

Similar experiments are conducted for Scenario 2 using the same parameters as for Scenario 1. Results are given in Figure 20. From Figure 20a it can be seen that in the case of N=1, with the number of jammers increasing, the number of “big” eigenvalues increases and keeps the same with the number of jammers. (Here, the word “big” denotes the situation when the eigenvalues are far larger than the ones representing the noise. Specifically in this experiment, it means the eigenvalues are more than 40 dB.) As shown in Figure 20d, in the case of N=6, the number of “big” eigenvalues is 3 for the first jammer and 6 for each of the next three jammers, corresponding exactly to the filter tap length. The number of “big” eigenvalues for the first jammer is less than the other three because the bandwidth of the last three jammers occupies the whole bandwidth of the B3 signal, while the bandwidth of the first jammer is half of the other three. This verifies that the DOFs consumed have a relationship with the bandwidth of the interference. Figure 20b,e show that the changes of the updating weight vector’s Eucidian-2 norm are in a relation to the number of jammers in the environment. The 2-norm of the weight vector increases with the number of jammers in SAP, while in STAP the relation is not that obvious. Figure 20c,f show that C/N0 of each tracking channel has a possibility of both increasing and decreasing when a new jammer is turned on. This is due to the distortion of the antenna pattern. Since each jammer is much weaker as compared to Scenario 1, the wideband nature of the jammers is less detrimental to SAP, making the SAP and STAP comparison more similar.

**Figure 21:**
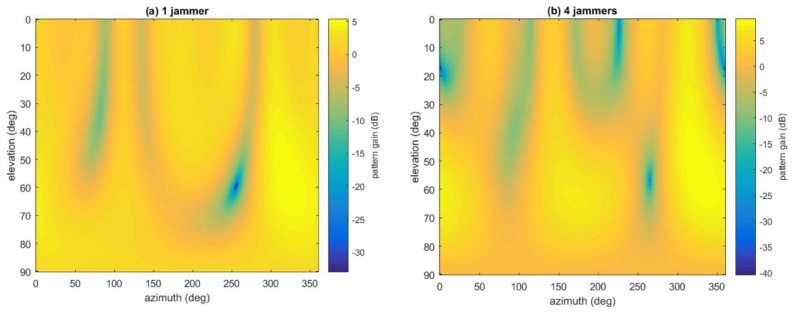
Antenna pattern of SAP nulling in Jamming Scenario 2.

Besides, given the weight vector and the geometry of the array, the antenna radio pattern can also be plotted roughly. Figure 21 gives two examples when SAP is used with the presence of one and four jammers, respectively, in Jamming Scenario 2. It can be seen that the pattern is very rough, because all the non-ideal factors of the antenna array and RF channels are ignored, including position inaccuracy of the array geometry, mutual coupling between antenna elements, finite ground-plane effect, mismatches between RF channels, different delays of cable lines, *etc.* [17,18]. Thus the plot is not so accurate and can only be used as auxiliary reference.

### 6.3. SFAP Nulling Experiments

**Figure 22:**
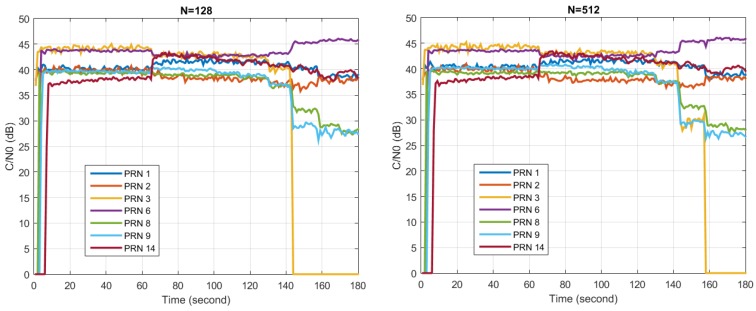
Performance of the SFAP nulling during Jamming Scenario 1. (**Left**) Results of C/N0 when N is 128; (**Right**) Results of C/N0 when N is 512.

For SFAP, similar experiments as presented in Section 6.2 can be conducted, in which eigenvalues of the covariance matrix and the updating weight vector Euclidian 2-norm can be analyzed in a similar manner for each frequency bin. The results and interpretations are similar to those in STAP, so these are not presented here in detail. However, as an example, the C/N0 of each tracking channel with different DFT lengths in Scenario 1 is presented in Figure 22. The antenna element number was set to 8, the overlap parameter was set to 0.4, while the number of snapshots taken to estimate the covariance matrix was set to 100. Diagonal loading was performed, where $\alpha_{dl}$ was set to 1 × 10^−6^. Two tests were carried out, with the DFT length set to 128 and 512, respectively. It can be seen that the anti-jamming performance with N=512 is slightly better than that with N=128, since at about second 150 the tracking channel of PRN 3 is still locked in the right figure, while it has already lost tracking in the left figure. However, this performance improvement is not obvious.

Comparing the results presented in Figure 19 and Figure 22, it can be seen that more satellites are lost in the STAP than in SFAP approach. The performance gain is due to that the spectrum resolution in the filtering behind each antenna in SFAP (corresponding to the filter tap length) is better than that in STAP (corresponding to the FFT length). According to [40], when the filter tap length in STAP and the FFT length in SFAP are equal, the signal-to-interference-plus-noise ratios (SINRs) obtained in the two approaches are the same. Since in our implementation, the FFT length in SFAP is significantly larger than the filter tap length in STAP, the performance gain is reasonable.

### 6.4. Beamforming Experiments

**Figure 23:**
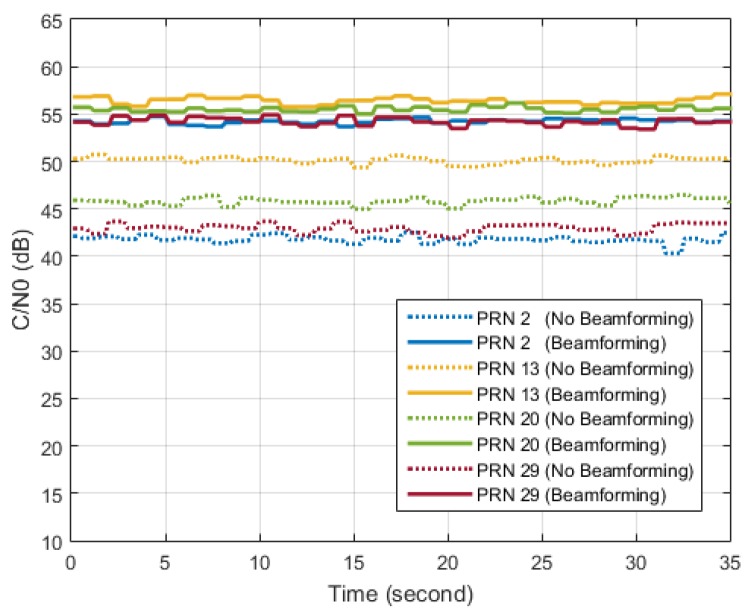
Results of C/N0 with beamforming (solid lines) and without beamforming (dotted lines).

In the last part of this section, results of a STAP beamforming experiment using the GPS L1 C/A signal are briefly introduced. Similar results can be obtained using the BDS B3 signal. In our implementation of beamforming, as many as 12 beams can be pointed to the visible satellites in the sky, but C/N0 of only 4 satellite signals are given in Figure 23 for brevity. C/N0 results without beamforming are also given as a comparison. It can be seen that considerable gains in C/N0 can be obtained by beamforming.

**Figure 24:**
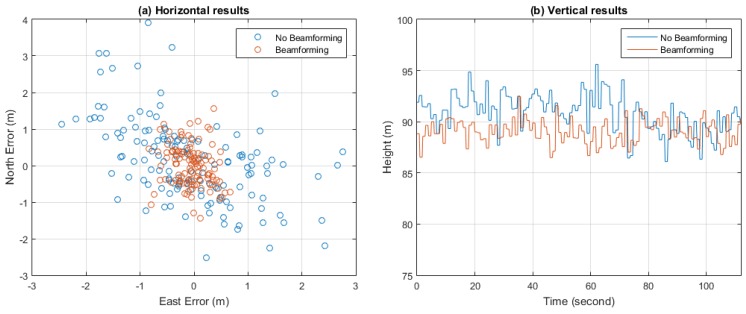
Positioning results with beamforming (red circles in graph-a) and red lines in graph-b) and without beamforming (blue circles in graph-a and blue lines in graph-b). The north error and south error are with respect to the average values, both with beamforming and without beamforming, respectively. The results are updated once per second. (**a**) Horizontal results; (**b**) Vertical results.

Figure 24 depicts the positioning results both with and without beamforming. The position results were calculated once per second. Table 6 gives the average values and standard deviations of the positioning results. It can be seen that with beamforming, more accurate positioning results can be obtained, thanks to the gains in C/N0.

## 7. Conclusions

This paper documents the design and implementation of an SDR-based real-time testbed for GNSS array anti-jamming. This platform is able to perform both STAP and SFAP algorithms to multi-element raw data at a sampling frequency of 20 MSPS, in either an adaptive nulling or a beamforming mode. When calculating the updating weight vector, either an SMI method in which the inversion of the covariance matrix needs to be calculated, or an iterative method such as LMS can be used. A wide range of parameters can be configured to explore their relation to the anti-jamming performance in specific interference scenarios. As examples, anti-jamming performances and the intermediate states of varied algorithms and parameters in certain jamming scenarios are given by experiments in real environments.

In order to meet the real-time requirement, a not so advanced GPU, *i.e.*, GTX 770, is used as an accelerator. Batched methods are proposed in the calculation of covariance matrices, the weight vectors as well as the outputs to make the best use of the parallelism of the computational resources provided by the GPU. Special attention is paid to provide a high level of configuration possibilities in the implementation. In the software architecture, besides the anti-jamming processing module, software receiver processing is also provided. Tests showed that the computational performance satisfies the real-time requirement very well.

This testbed significantly outperforms previous similar systems in function, flexibility, configurability as well as computational performance. Besides, as far as the authors know, this is the first time a real-time implementation of SFAP and the SMI method has been realized using a software-defined approach in GNSS array anti-jamming field in open literature. It is hoped that this testbed can be used as a fertile ground for researchers to test and validate different adaptive array anti-jamming algorithms in various jamming scenarios. It can also be used for product prototyping, and even plays a role in certain applications like signal monitoring in challenging environments.

## Figures and Tables

**Table 1:** Overview of previous work and testbed presented in this paper.

Work	Jiwon Seo (Stanford) [7]	Yu-Hsuan Chen (Stanford) [8]	This Paper
Anti-jamming mechanism	Adaptive beamforming only	Adaptive beamforming only	Both adaptive nulling and beamforming
Algorithm types	SAP	STAP	SAP, STAP and SFAP
Configurability	Limited	Limited	Parameters can be configured in a wide range
Weights calculation method	A propagating (iterative) approach. No inverse calculation of the covariance matrix	A propagating (iterative) approach. No inverse calculation of the covariance matrix	The SMI approach
Antenna element number	4 antenna elements	4 antenna elements	Configured antenna element number, which is 8 in maximum
Process samples	14-bit resolution samples at a rate of 40 MSPS (20 MSPS inphase and quadrature samples)	16-bit resolution samples at a rate of 40 MSPS (20 MSPS inphase and quadrature samples)	14-bit resolution samples at a rate of 40 MSPS (20 MSPS inphase and quadrature samples)
Processing Core	Intel Core i7 950 CPU and a NVDIA GTX 480 GPU	An x86-based multi-core CPU	An x86-based multi-core CPU and a NVDIA GTX 770 GPU
Application	For anti-jamming reference station receivers	For anti-jamming stationary receivers	For researching, prototype testing, as well as anti-jamming stationary receivers

**Table 2:** Configuration parameters of the Spatial-time adaptive processor (STAP) and their value range.

Parameter	Meaning	Value Range
M	Antenna element number	4, 5, 6, 7, 8
N	Filter tap number	1 to 10
K	Number of snapshots taken to estimate the covariance matrix	100 to 1000
$N_{ref}$	Reference tap index	1 to N
$\sigma_{dl}$ or $\alpha_{dl}$	Diagonal loading factor	Positive small number

**Table 3:** Configuration parameters of the Spatial-frequency adaptive processor (SFAP) and their value range.

Parameter	Meaning	Value Range
M	Antenna element number	4, 5, 6, 7, 8
N	DFT length	64, 128, 256, 512, 1024
K	Number of snapshots to estimate the covariance matrix	32 to 500
$\sigma_{dl}$ or $\alpha_{dl}$	Diagonal loading factor	Positive small number
Window type	Function to window the time domain samples	Rectangle, Triangle, Bartlett, Hann, Hamming, Blackman, *etc.*[22]
overlap	Overlapping ratio	Non negative number less than 1

**Table 4:** Thread number of *gpu_Rxx_batched* per block for covariance matrices of different sizes.

Matrix Size	Tile Count	Thread Number
4×4 to 29×29	1	16 to 841
30×30 to 59×59	3	225 to 900
60×60 to 80×80	6	400 to 729

**Table 5:** Information of the 4 jammers in Scenario 2.

Jammer	Bandwidth	Start Time
Jammer 1	10 MHz	Second 32
Jammer 2	20 MHz	Second 51
Jammer 3	20 MHz	Second 69
Jammer 4	20 MHz	Second 84

**Table 6:** The average values and standard deviations of the positioning results.

Statistic Character		Beamforming	No Beamforming
Average positioning results	Longitude (°)	116.330340	116.330347
	Latitude (°)	40.001468	40.001470
	Height (m)	89.03	90.52
The standard deviations of the positioning results with respect to the average values	East error (m)	0.32	1.05
	North error (m)	0.57	1.13
	Height (m)	1.17	1.86

## Appendix A

In the implementation of STAP on the GPU, many weight vectors are calculated using the Choleksy factorization method concurrently by the blocks in one kernel. The following table presents the algorithm of the Choleksy solver for each block.

**Algorithm 1: Cholesky Solver for Each Block**	
	*%* tdx *denotes the thread index within the block* *% At first,* R *is saved in* A *and* s *is saved in* h *% Line 1 to 20 denote the Cholesky factorization and the forward iteration*
1:	IF tdx=0 THEN
2:	$A(0,0)=\sqrt{A(0,0)}$
3:	$h(0)=\frac{A(0,0)}{h(0)}$
4:	END IF
5:	syncthreads()
6:	IF tdx>0
7:	$A(tdx,0)=\frac{A(tdx,0)}{A(0,0)}$
8:	END IF
9:	syncthreads()
10:	FOR i=1:MN−1
11:	IF tdx=i
12:	$A(i,i)=\sqrt{A(i,i)-\sum_{j=0}^{i-1}|A(i,j){|}^{2}}$
13:	$h(i)=\frac{1}{A(i,i)}[h(i)-h(j)A(j,i)]$
14:	END IF
15:	syncthreads()
16:	IF tdx>i
17:	$A(tdx,i)=\frac{1}{A(i,i)}(A(tdx,i)-\sum_{j=0}^{i-1}(A(i,j)A{\left(tdx,j\right)}^{*}))$
18:	END IF
19:	syncthreads()
20:	END FOR
	*% Until now,* L *is saved in* A *and* d *is saved in* h *% Line 21 to 27 denote the backward iteration*
21:	IF tdx=MN−1
22:	$h(MN-1)=\frac{h(MN-1)}{A{\left(MN-1,MN-1\right)}^{*}}$
23:	END IF
24:	syncthreads()
25:	FOR i=MN−2:−1:0
26:	$h(i)=\frac{1}{A{\left(i,i\right)}^{*}}(h(i)-\sum_{j=i+1}^{M-1}h(j)A{\left(j,i\right)}^{*})$
27:	END FOR *% In the end,* b *is saved in* h
